# The effects of the changes in the depression on suicidal ideation among older adults aged 75 and above before and after the COVID-19

**DOI:** 10.1186/s12877-024-05427-x

**Published:** 2024-10-24

**Authors:** Kyu-Hyoung Jeong, Sunghwan Cho, Yeon Jae Hwang, Dayoon Park, Seoyoon Lee

**Affiliations:** 1 Department of Social Welfare, Jeonbuk National University, 567 Baekje-Daero, Jeonju, 54896 Republic of Korea; 2 School of Social Welfare, The Catholic University of Korea, 43 Jibong-Ro, Bucheon-Si, Gyeonggi-Do 14662 Republic of Korea; 3 Department of Social Welfare, University of California, Los Angeles, 337 Charles Young Dr E, Los Angeles, CA 90095-1656 USA; 4 Korea Central Public Agency for Social Service, 340, Samil-daero, Jung-Gu, Seoul, Republic of Korea; 5 Department of Health Policy and Management, School of Public Health, Texas A&M University, 212 Adriance Lab Rd, College Station, Texas USA

**Keywords:** Depressive Symptom, Aged, COVID-19, Republic of Korea

## Abstract

**Background:**

The unprecedented pandemic situation of COVID-19 has had a negative impact on the mental health of many people, especially among the "old-old" older adults who are aged 75 or older. Therefore, the aim of this study is to investigate the changes in depression among "old-old" older adults before and after the onset of COVID-19, and the extent to which depression affects suicidal ideations.

**Method:**

The 12th to 16th Korea Welfare Panel Study(KoWePS) conducted from 2017 to 2021 was used for analysis. For this study, 771 older individuals with complete data to estimate the degree of change of depression were selected as the final analysis subjects.

**Result:**

A Growth Mixture Modeling(GMM) analysis was conducted, resulting in the classification of two groups: an increasing group and a decreasing group. The study findings showed that "old-old" older individuals with high levels of depression, specifically those in the decreasing group, may be more susceptible to suicidal ideation. Despite this steep change slope, the decreasing group still exhibited a higher level of depression in 2021 compared to the increasing group. As per characteristics, the decreasing group, which showed a higher prevalence of suicidal ideation, had a higher proportion of women and individuals with lower levels of education, those living alone, and a lower household income compared to the increasing group.

**Conclusion:**

It is important to note that although the study emphasized the need to prioritize intervention for the decreasing group with consistently high levels of depression, the majority of individuals belong to the increasing group, which exhibited a gradual increase in depression levels over time. Therefore, intervention plans should be developed concurrently for both groups. Also, it is crucial to implement proactive efforts targeting groups with understandings of these characteristics when establishing preventative measures for depression and suicidal ideation among "old-old" older adults.

## Background

The emergence of the COVID-19 virus towards the end of 2019 brought about significant changes not only in the social and economic landscape of the country but also in the daily routines of numerous individuals. The implementation of lockdowns across the globe led to the social isolation of a segment of the population who experienced job loss as a consequence of the employment shock. The alterations brought about by the COVID-19 pandemic have had an adverse impact on mental health [18]. In fact, several countries have reported a significant rise in anxiety and depression levels since the outbreak gained momentum in 2020 [29]. Since the outbreak of the COVID-19 pandemic, the mental health of the Korean population has undergone significant transformations. As of June 2022, the proportion of individuals at risk of developing depression was 16.9%, which is over five times higher than the corresponding figure recorded in 2019, prior to the pandemic [24]. The prevalence of the "Corona Blue" phenomenon, a combination of COVID-19 and depressive feelings [35], has intensified as the pandemic persists, making it imperative to implement proactive measures at the societal level to prevent mental health deterioration.

The risk of suicide and suicidal thoughts is escalating along with the surge in depression due to COVID-19. Depression is one of the most prevalent mental illnesses among individuals with suicidal ideations [9]. Given that depression is a significant predictor of suicide, the growing cases of depression induced by the COVID-19 pandemic could lead to severe societal consequences, including suicide. According to the Korean National Mental Health Survey on COVID-19, conducted in June 2022, the rate of suicidal ideation in Korea was found to be 12.7%, which is nearly three times higher than the corresponding figure recorded in 2019 [24]. Given that South Korea’s suicide rate is more than twice the OECD average [27], these figures suggest that South Korea is at high risk of further deterioration in mental health post-COVID-19.Therefore, there is a dire need for academic attention to improving the mental health of the population during the COVID-19 pandemic, and a specific exploration of depression, which is one of the primary predictors of suicide.

The unprecedented pandemic situation of COVID-19 has had a negative impact on the mental health of many people, especially among the older population. The risk of severe illness or death is higher for older people when they are infected with COVID-19 [21]. In addition, unlike young people who maintain relationships with the outside world through social media or work, older adults are more likely to become socially isolated when community centers, welfare centers, and religious activities are prohibited due to COVID-19 [36]. Among them, older adults are expected to be more vulnerable to the COVID-19 pandemic, especially the so-called "old-old" older adults who are aged 75 or older [28]. The "old-old" older adults are generally more psychologically and socially vulnerable and have a higher risk of social isolation than “young-old" older adults, who are under 75 years old, due to chronic illnesses, physical disabilities, spousal death, and the death of significant others [32]. Additionally, there is a higher possibility that mental health issues, such as depression, may be relatively more vulnerable among old-old individuals compared to young-old individuals [10, 22]. Actually, in Korea's 2020 age-specific suicide rate, The suicide rate among those aged 80 and over was 62.6 per 100,000, the highest among all age groups. This is also about three times higher than the OECD average [27]. It has also been confirmed that the severity of depression symptoms increases with age, with older adults aged 85 and above showing three times higher depression symptoms (24.0%) than those aged 65 to 69 (8.4%) [25]. Therefore, social welfare research and academic discussion should focus on depression and suicide problems among late older adults after COVID-19.

The previous findings that depression and suicide issues among late older individuals are severe suggest that mental health problems in older adults may vary by age group. Accordingly, recent studies have attempted to analyze the changes in depression and their levels by age. These studies compared the factors affecting depression in the early and late older adults by distinguishing them [15, 22] or analyzed the changes in depression trajectories among older adults over time [1, 13, 20]. These studies are meaningful in that they have identified various changes in the trajectory of depression in old age and, through this, seek effective intervention methods and differentiated strategies tailored for each group. However, since the analysis subjects are focused on all ages of among older adults, there are limitations in specifically identifying the level of depression changes among older adults aged 75 and above. Accordingly, there were studies that examined the longitudinal trajectory of depression among ‘old-old’ older adults and its predictive factors [10, 38]. However, these studies have limitations in that they target only the oldest population (85 years or older) or do not analyze the relationship between depression and suicidal thoughts among the aging population. In other words, there is a lack of longitudinal studies specifically examining the patterns of depression and their relationship with suicide among late older individuals, who are relatively more vulnerable to mental health issues. Considering the recent ‘Corona Blue’ phenomenon due to the COVID-19 pandemic, it is even more important to analyze the changes in depression and suicidal thoughts in the elderly before and after COVID-19.

Therefore, this study aims to explore the changes in depression among ‘old-old’ older adults before and after COVID-19, as well as how depression trajectories affect suicidal ideation. In particular, considering that the depression trajectories of older adults can be classified into various types [1, 13], this study conducts a Growth Mixture Modeling (GMM) to explore the relationships between each group and these variables in a multi-layered manner. The GMM is useful for identifying heterogeneous subgroups within a population and explaining their change trajectories [12]. In other words, this study aimed to conduct a more multifaceted analysis of the types of depression changes in the aging population and the characteristics of each group, beyond the method of analyzing depression and suicide among older adults only in terms of their relationship with specific variables.

Through this study, we aim to analyze the psychological changes experienced by older adults, who are relatively vulnerable to depression and suicide, after the emergence of COVID-19. Additionally, in anticipation of the "post-COVID-19 era" due to the prolongation of infectious diseases, we tried to identify interventions that can prevent depression and suicide among "old-old" older adults. The specific research questions that this study seeks to answer are as below:

1. What are the types of changes in depression observed among "old-old" older adults before and after the COVID-19 pandemic?
2. What is the correlation between the types of depression and suicidal ideation among "old-old" older adults before and after the COVID-19 pandemic?

## Material and methods

This study aims to explore the relationship between suicidal ideation and the identification and classification of depression among the late older population. Therefore, in this study, the 12th to 16th Korea Welfare Panel Study (KoWePS) conducted from 2017 to 2021 was used for analysis. The Korea Welfare Panel Survey is a survey panel that represents the welfare situation in Korea. Its objective is to provide feedback to policymakers on issues such as the development of new policies and institutional improvements by determining the living conditions and welfare requirements of different population groups and assessing the effectiveness of policy implementation. The Korean Welfare Panel Survey (KoWePS) is a joint effort conducted by eight researchers from the Korea Institute for Health and Social Affairs (including one research fellow, four researchers, and three research assistants) and eight researchers from the Institute of Social Welfare at Seoul National University (including five professors, two doctoral students, and one master's student). The survey primarily uses direct face-to-face interviews, where researchers visit the panel households and record the responses of household members using Computer Assisted Personal Interviewing (CAPI). However, if researchers were unable to meet the respondents directly during the survey period due to inevitable reasons such as late-night returns or long-term absences, or if household members are not present due to reasons such as living abroad, travel, business trips, hospital admissions, or military service, telephone or proxy interviews were conducted on a limited basis. The survey was carried out annually from February to March. In this study, we focused on late older individuals (aged 75 and older), excluding early older individuals (aged 65–74). The final analysis included 771 participants, for whom changes in depression were estimated from 2017 (the 12th wave) to 2021 (the 16th wave).

### Variables

#### Independent variable: depression (2017–2021)

The study used the abbreviated version of the Center for Epidemiologic Studies Depression (CES-D) scale by Kohout et al. [16] as the independent variable to measure the level of depressive symptoms in a non-diagnostic way. The abbreviated version, which includes 11 items, was developed by Radloff [34] and Kohout to address the length of the original scale. Responses were scored on a 4-point scale (1 = very rarely, 2 = sometimes, 3 = often, 4 = mostly), with higher scores indicating higher levels of depression. In this study, an average point of 11 items was used. For reference, the CES-D developed by Radloff [34] consists of 20 items rated on a 0–3 point scale, with a cut-off score of 16 points for identifying depression. In contrast, the Korea Welfare Panel uses 11 items rated on a 1–4 point scale. This study utilized the average score of the 11 items to better understand changes in depression over time. Therefore, it is challenging to provide a cut-off point based on the existing total, and since the aim is not to confirm depression, we have excluded a reference point to avoid confusing readers. The reliability of the depression scale, as measured by Cronbach's α, was 0.886 in 2017, 0.872 in 2018, 0.869 in 2019, 0.845 in 2020, and 0.866 in 2021.

#### Dependent variable: suicidal ideation (2021)

The dependent variable for suicidal ideation was assessed using the question, "Have you had any suicidal thoughts in the past year?" Participants were scored as 0 if they reported no suicidal thoughts and 1 if they reported any suicidal thoughts.

#### Control variables: sociodemographic characteristics (2017)

The control variables in this study include various sociodemographic characteristics such as sex (with male coded as 0 and female coded as 1), age (a continuous variable), equalized annual income (a continuous variable), education level (with elementary school or lower coded as 0 and middle school or higher coded as 1), area of residence (with urban coded as 0 and suburban and rural coded as 1), and living with someone (living with someone coded as 0 and living alone coded as 1). Notably, the equalized annual income was calculated by dividing the household annual income by the square root of the number of household members, and the logarithm was transformed for normal distribution.

### Statistical analysis

In this study, Stata 15.1 and M-plus 8.0 programs were used for data analysis. The analysis method and procedure included several steps. First, descriptive statistical analysis was conducted to examine the sociodemographic characteristics and main variables of the analysis target. Second, a Latent Growth Model was performed to estimate the overall change in depression assuming a single group. Model fit was evaluated using TLI (Tucker-Lewis Index), CFI (Comparative Fit Index), and RMSEA (Root Mean Square Error of Approximation), with simplicity of the model being prioritized over sensitivity to sample size. Third, a Growth Mixture Modeling (GMM) was conducted to determine the type of change in depression. While Latent Growth Model (LGM) is an analysis that assumes that the population is identical, but GMM relaxes this single population assumption and acknowledges that there may be subpopulations with different characteristics within the entire population [26]. GMM estimates the number of unobserved latent groups from repeated measurement longitudinal data and identify different trajectories for each group and the factors explaining each trajectory [6]. The number of latent classes in a GMM is determined by starting with a single-class model, similar to the LGM results, and then incrementally increasing the number of classes to analyze up to a k-class model. The appropriate number of classes (k) is chosen based on model fit indices (such as Information Criteria: IC), the sample proportions within each subpopulation, and considerations of the research question or theoretical rationale.

The optimal number of change types was determined using the *p*-values of AIC (Akiakie's Information Criteria), BIC (Bayesian Information Criteria), SSABIC (Sample-Size Adjusted BIC), Entropy, and BLRT (Bootstrapped Likelihood Ratio Test). Finally, chi-square analysis and independent sample t-test were conducted to confirm the difference in demographic characteristics according to the type of change in depression. Fourth, we conducted logistic regression analysis using the Firth method to examine the relationship between the type of change in depression and suicidal ideation. Conventional logistic regression analysis may produce biased estimates of coefficients when dealing with variables such as suicidal ideation where only a small number of cases account for the majority of cases without the outcome of interest [14]. To address this issue, this study used the penalized maximum likelihood estimation method proposed by Firth [8], which allows for unbiased coefficient estimates and is suitable for rare events. This approach can improve the accuracy of the analysis and the reliability of the results [7].

## Results

### Descriptive statistics

The demographic characteristics of the study participants are presented in Table 1. In terms of sex, there were 265 men (34.4%) and 506 women (65.6%), indicating a higher proportion of women than men. The mean age was 77.99 years (Standard Deviation [SD] = 2.90) and the equivalent household income was USD $10,524.08 (SD = 6,489.94). In terms of education, 564 participants (73.2%) had an elementary school education or lower, while 207 (26.8%) had a middle school education or higher. Regarding area of residence, the number of participants living in urban area was 243 (31.5%), which was lower than the number of participants living in suburban or rural area (528, 68.5%). In terms of living arrangements, 403 participants (52.3%) were living with someone, which was similar to the number of participants living alone (368, 47.7%). With respect to suicidal ideation, 752 participants (97.5%) reported never having had suicidal thoughts, while 19 participants (2.5%) reported having had such thoughts at least once.

**Table 1:** Sociodemographic characteristics of the study participant (*N* = 771)

**Variable**	**Categories**	**N**	**%**
Sex	Men	265	34.4
	Women	506	65.6
Age (M (SD))		77.99(2.90)	
$ Equivalent Household Income (M (SD))		10,524.08(6,489.94)	
Education	Below Elementary School	564	73.2
	Above Middle School	207	26.8
Area of Residence	Urban	243	31.5
	Suburban and rural	528	68.5
Living Arrangement	Living with Someone	403	52.3
	Living Alone	368	47.7
Suicidal Ideation	No	752	97.5
	Yes	19	2.5

The descriptive statistics of the main variable, depression, showed an increasing trend over time (Table 2), with a mean depression score of 1.51 (SD = 0.50) in 2017 and 1.67 (SD = 0.51) in 2021.

**Table 2:** Descriptive statistics of the main variable (Depression)

Variable	Min	Max	M	SD
Depression-2017	1.00	3.55	1.51	.50
Depression -2018	1.00	3.91	1.53	.47
Depression -2019	1.00	3.45	1.54	.48
Depression -2020	1.00	3.64	1.58	.47
Depression -2021	1.00	4.00	1.67	.51

*Min.* Minimum, *Max* Maximum, *M* Mean, *SD* Standard Deviation

### Types of depression changes

Prior to implementing a GMM, a Latent Growth Model was conducted to assess the overall change in depression. The no-growth model and linear growth model were separately analyzed and compared based on their model fit. The findings indicated that the linear growth model (χ^2^ = 37.010 (*p* < 0.001), CFI = 0.958, TLI = 0.958, RMSEA = 0.059) outperformed the no-growth model in meeting the fitness criteria (Table 3). Consequently, the linear growth model was selected as the ultimate model.

**Table 3:** Estimating the model fit for changes in depression in latent growth modeling

Model	χ^2^	CFI	TLI	RMSEA
No-growth model	130.412^***^	.815	.858	.108
Linear growth model	37.010^***^	.958	.958	.059

^***^ *p* < .001

Based on the linear growth model, a GMM was estimated to identify the types of depression change (Table 4). The fitness of each model was compared, and it was found that the models with 1, 2, and 4 groups of classification had higher AIC, BIC, and SSABIC values than the model with 3 groups. The 2-group classification was found to have Entropy closer to 1 than other types. However, since there was a group that accounted for less than 5% of the total cases in the 3 groups and 4 groups classifications, the final decision was made to adopt the 2-group classification.

**Table 4:** The fitness of the Model for GMM (*N* = 771)

Class	Model fit					Groups
	AIC	BIC	SSABIC	Entropy	BLRT *p*-value	n(%)
1	4801.791	4866.859	4822.402	-	-	-
2	4687.917	4748.337	4707.056	.884	< .001	704(91.3), 67(8.7)
3	4628.986	4703.349	4652.542	.792	< .001	613(79.5), 120(15.6), 38(4.9)
4	4634.986	4723.292	4662.958	.835	< .001	613(79.5), 120(15.6), 20(2.6), 18(2.3)

**Fig. 1:**
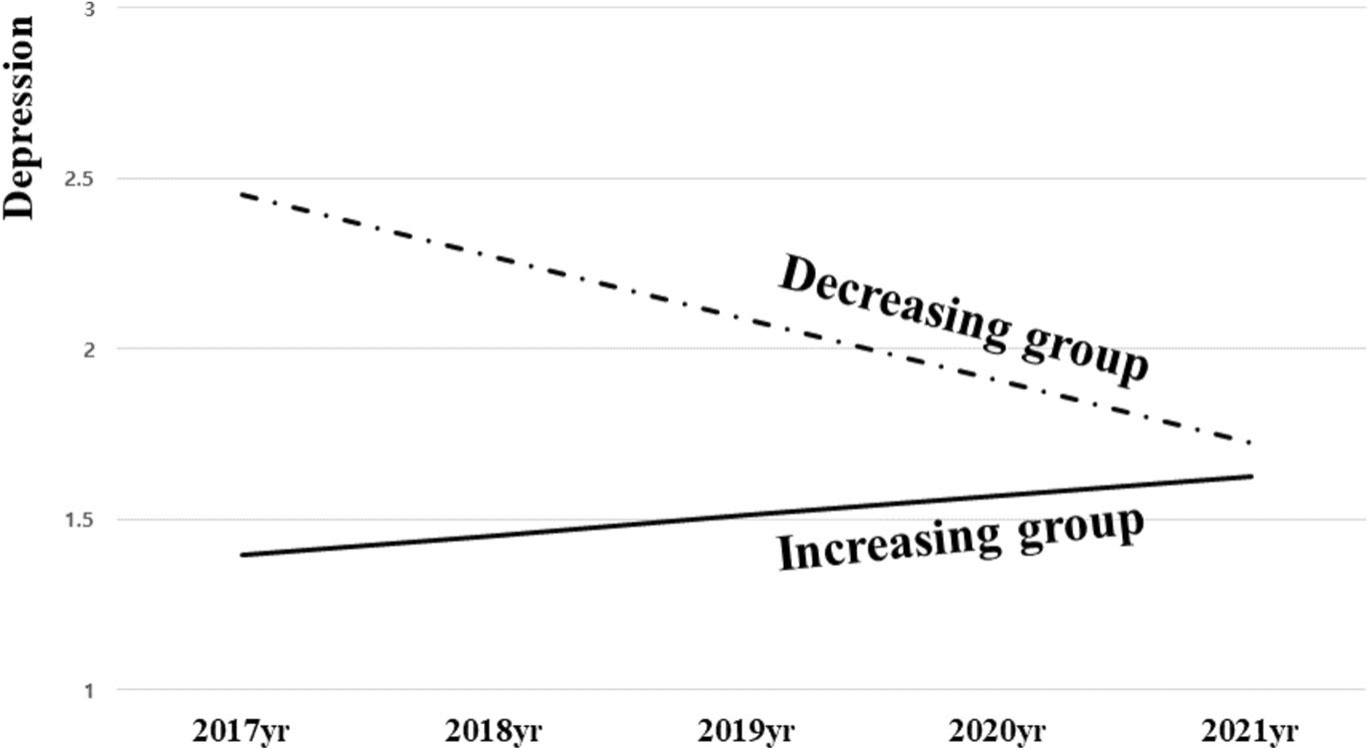
Estimation of the type of depression changes among 'old-old' older adults

Two types of depression changes were identified and named based on their characteristic change patterns (Fig. 1). The first type was named the "increasing group," which exhibited a trend of depression increasing from 2017 to 2021. This type included 704 cases (91.3%). The second type was named the "decreasing group," which showed a continuous decrease in depression from 2017 to 2021. This type included 67 cases (8.7%).

We conducted a chi-squared analysis and independent t-tests to examine the differences in sociodemographic characteristics according to the groups of depression changes (Table 5). The analysis revealed that the increasing and decreasing groups of depression changes showed differences in terms of sex (χ^2^ = 14.261, *p* < 0.001), Equivalent Household Income (t = 4.559, *p* < 0.001), education level (χ^2^ = 8.303, *p* < 0.01), and living alone (χ^2^ = 7.958, *p* < 0.01). Regarding sex, the increasing group had a higher proportion of men than the decreasing group, while the increasing group had a higher Equivalent Household Income. In terms of education level, the decreasing group had a higher proportion of people with elementary school or lower education than the increasing group, and regarding living alone, the decreasing group had a higher proportion of people living alone than the increasing group.

**Table 5:** Demographic differences according to types of depression changes in the “old-old” older adults

Variable	Categories	Types of Depression Changes				χ^2^/t
		Increasing group		Decreasing group		
		N	%	N	%	
Sex	Men	256	36.4	9	13.4	14.261^***^
	Women	448	63.6	58	86.6	
Age (M (SD))		77.97(2.94)		78.13(2.50)		-.438
$ Equivalent Household Income (M (SD))		10,760.67(6,605.67)		8,038.10(4,443.34)		4.559^***^
Education	Below Elementary School	505	71.7	59	88.1	8.303^**^
	Above Middle School	199	28.3	8	11.9	
Area of Residence	Urban	219	31.1	24	35.8	.630
	Suburban and rural	485	68.9	43	64.2	
Living Arrangement	Living with Someone	379	53.8	24	35.8	7.958^**^
	Living Alone	325	46.2	43	64.2	

^**^p < .01

^***^p < .001

### Relationship between depression changes and suicidal ideation

The Firth Method was utilized to examine the correlation between changes in depression and suicidal ideation (Table 6). The statistical model was determined to be significantly fitting (χ^2^ = 58.33, *p* < 0.001). The findings of the analysis demonstrated that the depression change type (Coefficient [Coef.] = 1.668, *p* < 0.001), as well as the controlling variable of residence (Coef. = -0.981, *p* < 0.05), were noteworthy determinants of suicidal ideation. Specifically, the study found that the probability of having suicidal ideation increases if the residence is in a urban area rather than a suburban and rural area and the depression change type is decreasing rather than increasing. In contrast, sex, age, income level, education, and living arrangements were not found to be significant determinants of suicidal ideation.

**Table 6:** Analysis of factors influencing suicidal ideations (*N* = 771)

Variables	Coef	S.E
Sex (ref. men)	-.533	.574
Age	.113	.064
Equivalent Household Income (log)	-.061	.472
Educational level	-.044	.591
Area of Residence (ref. Urban)	-.981^*^	.476
Living Arrangement (ref. Living with someone)	.339	.541
Types of changes in depression (ref. Increasing group)	1.668^***^	.807
constant	-10.964	6.799

^*^p < .05

^***^p < .001

## Discussion

This study aimed to investigate the relationship between depression changes over time and suicidal ideation among "old-old" individuals in Korea. To achieve this, the responses of 771 participants were analyzed using data from the Korean Welfare Panel Study conducted between 2017 and 2021. The following are the key research findings and discussions.

Firstly, it was found that the level of depression among Korean "old-old" older adults gradually increased over time. This result is consistent with previous studies reporting that depression levels increase as people age [3, 30], suggesting the need for systematic mental health measures for entering a new stage of life in old age. In addition, since the results of this study show that depression tends to deepen as people age, continuous mental health management strategies should be developed for those who have already entered old age. Specifically, based on the results of this study, there is a high possibility that the level of depression in older late-life older adults would be the most severe, therefore, it will be necessary to concentrate on monitoring this group and providing detailed interventions according to their characteristics in early, middle, and late stages.

Secondly, to determine the different types of depression changes among older individuals, a GMM analysis was conducted, resulting in the classification of two groups: an increasing group and a decreasing group with a continuous decline. The increasing group, which consisted of most of the study participants, demonstrated a consistent increase from the initial survey in 2017 to the final survey in 2021. There were, however, only minor increases in depression levels overall. In contrast, the decreasing group began at a high level of depression in 2017, unlike the increasing group, and underwent a rapid decline, resulting in a significant gap between the initial and 2021 values. Despite this steep change slope, the decreasing group still exhibited a higher level of depression in 2021 compared to the increasing group (Fig. 1).

Thirdly, examining the relationship between the two groups of depression changes and suicidal ideation revealed that the probability of having suicidal ideation was greater for the decreasing group than for the increasing group. This finding indicates that, despite the fact that the depression level of the older people in the decreasing group decreased rapidly over time, their average level of depression during the observed period was consistently higher than that of the increasing group. These results can be primarily explained through previously reported associations between depression and suicidal ideation. Numerous studies have shown that depression in older adults is a significant cause of suicidal ideation [2, 4, 33] and plays a pivotal role in leading to negative outcomes such as suicide attempts [19, 23, 39]. Therefore, the higher probability of suicidal ideation observed in the decreasing group compared to the increasing group in our study may be attributed to this group's consistently higher depression levels.

Additionally, our findings related to suicide ideation may stem from the distinct characteristics of the decreasing group. This group exhibited a higher prevalence of suicidal ideation compared to the increasing group. It included a higher proportion of women, individuals with lower education levels, those living alone, and those with lower household income than the increasing group. Our results align with previous studies that have identified gender [17], older age [31], education level [40], living alone [37], and low income [11] as major risk factors for suicidal ideation in old age. Consequently, it is crucial to conduct systematic and regular surveys and implement proactive efforts targeting groups with these characteristics when establishing preventative measures for depression and suicidal ideation among "old-old" older adults.

Overall, the study findings showed that "old-old" older individuals with high levels of depression, specifically those in the decreasing group, may be more susceptible to suicidal ideation. Based on our findings, when devising preventative measures for depression in old age, it is crucial to utilize the accumulated data from monitoring the depression levels of old-old older adults to implement tailored interventions that match changes in depression levels. Our study indicates that special attention is needed in providing mental health measures for the decreasing group as they still showed higher levels of depression compared to the increasing group, as well as significant association with suicide ideation. However, this does not mean that measures for the increasing group should be overlooked since, despite their slight increase in depression, they exhibited a continuous upward trend over time, and the majority of individuals belong to the increasing group. Thus, it is crucial to take a nuanced approach, considering the varying trajectories of depression among different groups of old-old older adults for effective prevention and intervention strategies.

One of the examples to consider is the University of Washington's Health Prevention Research Center (HPRC) PEARLS intervention program. HPRC researchers provided long-term home visits from community social workers and professional psychological counseling for older adults with mild depression, and as a result, the depression levels of the experimental group were reduced by about 50%, and even 36% were found to have completely relieved depression symptoms [5]. As this program was conducted for low-income older adults, it is necessary to consider developing life-cycle tailored programs for both the decreasing and increasing groups and introducing community-based depression management systems by referring to this program.

## Conclusion

This study explored the longitudinal prevalence of depression among Korean "old-old" individuals and examined the patterns of changes in depression over a period of five years. In the process, the types of depression in "old-old" individuals were identified, and a significant relationship between suicidal ideation and depression was confirmed, distinguishing it from previous research. Furthermore, the study provided implications for the importance of intervention for specific groups of older individuals and the direction of possible intervention plans based on the research findings. However, this study has limitations in that it could not control for the external influence of the COVID-19 pandemic on the changes in depression levels of older adults and the limitations in setting various control variables due to the secondary data used. Therefore, future follow-up studies that supplement these limitations are expected. Finally, since the suicidal ideations variable used in this study is a nominal variable, it may be challenging to validate its accuracy. Therefore, we recommend using validated Likert scales for suicidal ideations in future research.

## Data Availability

The datasets generated and/or analysed during the current study are available at the Korea Welfare Panel Study (KoWePS) website: https://www.koweps.re.kr:442/main.do;jsessionid=976096DC561B558CA80613A33DCE4EA9.
